# Integrative Metabolomics and Whole Transcriptome Sequencing Reveal Role for TREM2 in Metabolism Homeostasis in Alzheimer’s Disease

**DOI:** 10.1002/alz.085147

**Published:** 2025-01-03

**Authors:** Meng Wang, Qi Qin, Yi Tang

**Affiliations:** ^1^ Department of Neurology & Innovation Center for Neurological Disorders, Xuanwu Hospital, Capital Medical University, Beijing, Beijing China; ^2^ Innovation Center for Neurological Disorders and Department of Neurology, Xuanwu Hospital, Capital Medical University, Beijing China; ^3^ Department of Neurology & Innovation Center for Neurological Disorders, Xuanwu Hospital, Capital Medical University, National Center for Neurological Disorders, Beijing, Beijing China; ^4^ Innovation Center for Neurological Disorders, Department of Neurology, Xuanwu Hospital, Capital Medical University, National Center for Neurological Disorders, Beijing China; ^5^ Neurodegenerative Laboratory of Ministry of Education of the People’s Republic of China, Beijing, Beijing China

## Abstract

**Background:**

Alzheimer’s disease (AD) is a progressive neurodegenerative disorder and the most common cause of dementia worldwide. Dysregulation of various metabolism pathways may mediate the development of AD pathology and cognitive dysfunction. Variants of triggering receptor expressed on myeloid cells‐2 (TREM2) are known to increase the risk of developing AD. TREM2 plays a role in AD development by maintaining cellular energy and biosynthesis, but the precise mechanism through which it accomplishes this is unknown.

**Method:**

In the present study, we explored whether TREM2 plays a role in AD development by maintaining cellular energy and biosynthesis with the help of an AD mouse model (APP/PS1). First, we performed untargeted metabolomics analysis of hippocampal tissues from APP/PS1 transgenic AD mice (APP/PS1‐TREM2 KO mice) and APP/PS1 transgenic AD mice (APP/PS1 mice), which revealed the changes of different metabolites after TREM2 knockdown. Next, we performed transcriptomic analysis to identify differentially expressed genes (DEGs). Finally, we used metabolomics and transcriptomics analysis to screen for genes associated with lipid metabolism.

**Result:**

Metabolomic analysis of hippocampal tissue from APP/PS1 and APP/PS1‐TREM2 knockout (KO) mice found that TREM2 KO was associated with abnormalities in several metabolism pathways, and the effect was particularly pronounced in lipid metabolism and glucose metabolism pathways. Consistently, transcriptomic analysis of these mice determined that most differentially expressed genes were involved in energy metabolism pathways. We screened seven differentially expressed genes in APP/PS1‐TREM2 KO mice that may influence AD development by altering energy metabolism. Integrative analysis of the metabolomic and transcriptomic profiles showed that TREM2 may regulate lipid metabolism and sphingolipid metabolism by affecting lipoprotein lipase (LPL) expression, thereby influencing AD progression.

**Conclusion:**

TREM2 knockout was found to be associated with abnormalities in multiple metabolic pathways in AD mice, particularly lipid metabolism and glucose metabolism. Also, LPL is a novel gene that may affect lipid metabolism and sphingolipid metabolism in AD mice.

